# The retinal nerve fibre layer thickness slope: a localised biomarker of the structure–function relationship in early glaucoma

**DOI:** 10.1136/bjo-2025-328330

**Published:** 2025-12-24

**Authors:** Stefan Steiner, Florian Frommlet, Florian Schwarzhans, Georg Fischer, Maximilian Pirrung, Michael Pircher, Christoph Hitzenberger, Clemens Vass

**Affiliations:** 1 Department of Ophthalmology and Optometry, Medical University of Vienna, Vienna, Austria; 2 Center for Medical Data Science, Medical University of Vienna, Vienna, Austria; 3 Research Center for Medical Image Analysis and Artificial Intelligence (MIAAI), Danube Private University, Krems an der Donau, Austria; 4 Center for Medical Statistics Informatics and Intelligent Systems, Section for Medical Information Management and Imaging, Medical University of Vienna, Vienna, Austria; 5 Center for Medical Physics and Biomedical Engineering, Medical University of Vienna, Vienna, Austria

**Keywords:** Glaucoma, Optic Nerve, Intraocular pressure, Visual pathway, Ocular Hypertension

## Abstract

**Background/aims:**

To describe the relationship between the novel biomarker retinal nerve fibre layer thickness slope (RNFL-S), and visual field sensitivity (VFS) in healthy and early glaucoma eyes.

**Methods:**

This prospective cross-sectional study of 50 early glaucoma and 139 healthy eyes analysed RNFL-S locally along retinal nerve fibre trajectories that were automatically traced and centred on 24–2 and 10–2 visual field (VF) test points. Corresponding virtual B-scans were extracted from stitched wide-field polarisation-sensitive optical coherence tomography images. A linear mixed-effects model (LMM) assessed the association between VFS and the factors RNFL-S, glaucoma status and age.

**Results:**

The average VF mean deviation was −3.11±1.51 dB in glaucoma (mean age: 63.0±9.3 years) and −0.36±1.10 dB in healthy eyes (mean age: 47.9±16.3 years). In healthy subjects, VFS and the corresponding RNFL-S were 31.8±2.4 dB (12.5±2.9 µm/mm) in the upper hemisphere and 32.4±2.2 dB (12.5±2.5 µm/mm) in the lower hemisphere. In glaucoma patients, these values were significantly lower: 27.5±7.0 dB (8.6 ± 3.9 µm/mm, p<0.001) in the upper hemisphere and 30.1±4.3 dB (10.5±3.3 µm/mm, p<0.001) in the lower hemisphere. Significant point-wise Spearman correlation coefficients (up to r=0.61) were observed, particularly in the upper VF hemisphere within the central 10° and close to the optic nerve head. The LMM showed a positive association between VFS and RNFL-S (β=0.0415, SE=0.0023, p<0.001, R²=0.1885). Glaucoma was significantly associated with lower VFS (β=−2.468, SE=0.069, p<0.001). Age negatively correlated with VFS (β=−0.048, SE=0.002, p<0.001).

**Conclusion:**

Local RNFL-S is significantly correlated with VFS, highlighting its potential as a biomarker for focal glaucoma damage.

**WHAT IS ALREADY KNOWN ON THIS TOPIC**

- The correlation between optical coherence tomography (OCT)-derived structural measures and visual field sensitivity (VFS) in glaucoma is still moderate. Despite the use of parameters such as retinal nerve fibre layer (RNFL) thickness, ganglion cell layer thickness and vessel density from OCT angiography, precise structure–function relationships remain difficult to establish. This limitation continues to challenge early and localised glaucoma detection.

**WHAT THIS STUDY ADDS**

- This study introduces the RNFL thickness slope (RNFL-S) along individual nerve fibre trajectories as a novel biomarker. RNFL-S reflects the local ganglion cell population and shows a significant local correlation with VFS in early glaucoma. It captures focal glaucomatous damage that may be overlooked by conventional structural measures.

**HOW THIS STUDY MIGHT AFFECT RESEARCH, PRACTICE OR POLICY**

- RNFL-S may serve as a biomarker for focal glaucomatous damage. Its use may enable improved structure–function mapping. Further research is needed to determine its potential clinical usefulness in glaucoma assessment.

## Introduction

 Glaucoma is a progressive optic neuropathy and a leading cause of irreversible blindness worldwide, characterised by structural damage to the optic nerve and corresponding functional deficits in the visual field (VF).^1^ Diagnosing glaucoma and monitoring disease progression relies heavily on the interplay between functional assessment and structural assessment. However, the relationship between structural biomarkers, such as optical coherence tomography (OCT)-derived measures, and functional outcomes, such as VF sensitivity (VFS), remains complex and only moderately correlated to date.^2^,^5^

The VF test is a subjective but indispensable tool for evaluating visual function in glaucoma. It provides direct insight into the ability of the patient to perceive visual stimuli, yet its variability and dependency on patient cooperation present challenges in longitudinal monitoring.^6^,^9^ OCT, on the other hand, offers objective, high-resolution imaging of retinal structures, including the retinal nerve fibre layer (RNFL) and ganglion cell layer (GCL), making it valuable for the diagnosis and monitoring of glaucoma.^10^,^12^ Despite the precision of OCT in detecting glaucomatous damage, predicting VFS solely from OCT parameters is still challenging.^23 13^,^15^ This highlights the need for better structural biomarkers that more closely reflect functional impairment.

The ganglion cells and their axons are key to visual processing, linking photoreceptors to higher visual pathways. Understanding how the integrity of GCL and RNFL relates to VFS is essential for improving glaucoma diagnostics. While prior studies have examined global and sectoral OCT-VF correlations, they often show only moderate associations and overlook localised structure–function relationships.^3 16^

Establishing causal relationships between VF defects and structural damage relies on accurate characterization of the trajectory of the retinal nerve fibre bundles (RNFBs). Manual tracing of nerve fibre bundles is tedious, impractical and therefore rarely used. To enable practical application in larger datasets, a mathematical model^17 18^ was developed to allow RNFB tracing and support the creation of individualised structure–function models. In the model introduced by Schwarzhans *et al*,^19^ additional structural information derived from polarisation-sensitive OCT was used to automatically trace RNFB trajectories.

In this study, we propose the local RNFL thickness slope (RNFL-S), measured along individualised RNFB trajectories, as a novel biomarker and indicator of the number of ganglion cell axons entering the RNFL, providing focal insight into visual function at specific retinal locations. By analysing local RNFL-S in relation to corresponding VF parameters, we aim to establish a more precise structure–function relationship and enhance the early diagnosis of glaucoma.

## Materials and methods

Participants in this study were part of the prospective cross-sectional ‘PS-OCT Fibre Tracing Study’.^20^ Subjects were recruited at the Center of Medical Physics and Biomedical Engineering and the Department of Ophthalmology and Optometry of the Medical University of Vienna. The study was approved by the local ethics committee and adhered to the tenets of the Declaration of Helsinki. Written informed consent was obtained from all subjects before participation.

Healthy participants were included if they were 18–79 years old, had a refractive error (spherical equivalent) between −5.0 and +5.0 D, a normal medical history (except for clinically irrelevant findings) and normal ophthalmic results, including a healthy optic nerve head (ONH) and VF.

Early glaucoma participants met the same general criteria but exhibited a glaucomatous ONH appearance (thinning or notching of the neuroretinal rim, or violation of the ISNT (Inferior > Superior > Nasal > Temporal) rule or cup-to-disc ratio >0.7 or asymmetry of cup-to-disc ratio >0.2), together with corresponding and consistently abnormal 24–2 VF results and a mean deviation (MD) better than −6.0 dB. The 24–2 VF tests were considered glaucomatous if they met the following criteria: at least three contiguous points on the pattern deviation probability plot with sensitivity reduced to p<0.05, including at least one point at p<0.01, confirmed on two consecutive VF tests no more than 2 weeks apart.

Exclusion criteria for both groups included a history of ocular trauma, ocular surgery within the past 3 months, any eye disease other than refractive error (unless clinically irrelevant), astigmatism ≥2.0 D, hypersensitivity to tropicamide or related agents, and pregnancy or breastfeeding.

In this study, we screened 197 healthy and 90 glaucoma participants, with one eye per participant. A total of 21 healthy and 12 glaucoma participants failed the screening process. Additionally, 6 healthy and 2 glaucoma participants were unable to complete the second study day due to COVID-19-related issues, and 2 healthy and 2 glaucoma participants withdrew from the study. Furthermore, 9 healthy and 2 glaucoma participants were excluded due to diabetes, which was not initially part of our exclusion criteria. Additional exclusions were related to image quality issues, including low signal strength or corneal compensation problems (3 healthy and 7 glaucoma), motion artefacts (4 healthy and 2 glaucoma), errors in the tracing or stitching algorithm (11 healthy and 12 glaucoma), and segmentation or trace path errors (2 healthy and 1 glaucoma). Ultimately, data from 139 healthy and 50 glaucoma participants were included in the final analysis.

**Figure 1:**
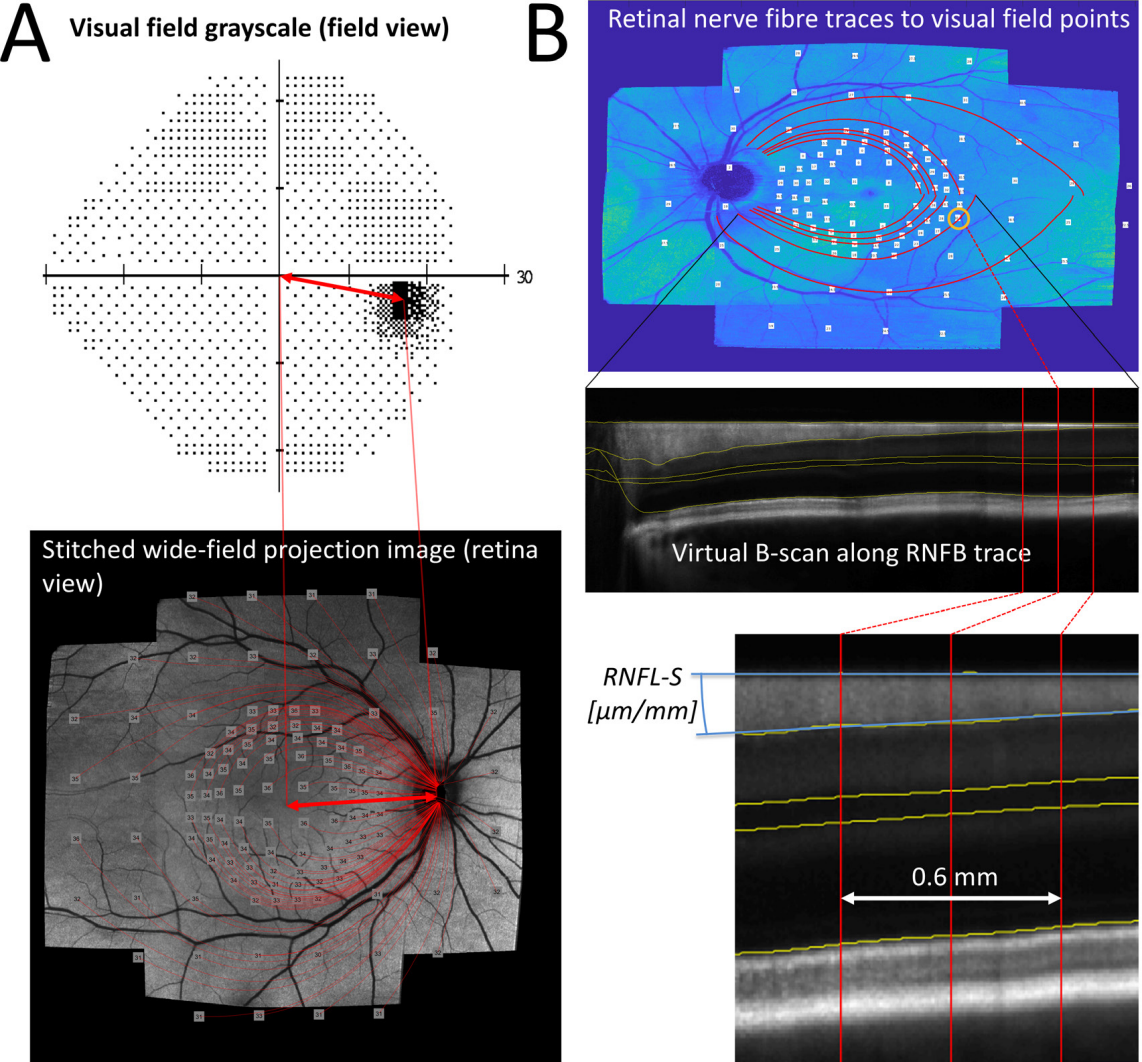
(A) Alignment of VF test points with the wide-field PS-OCT volume by flipping, scaling and rotating according to the blind spot and foveal coordinates, matched to the corresponding ONH and foveal centres. The red arrow indicates the distances between the fovea and the blind spot for the VF, and between the fovea and the ONH centre for the OCT volume, which were then used for scaling and rotation to match the VF with the OCT volume. VF test point positions were adjusted following the Drasdo *et al*^21^ model. (B) Virtual B-scan extraction along an individual RNFB to a VF test point and calculation of the RNFL-S over a 0.6 mm segment centred on the exemplary target VF point. ONH, optic nerve head; PS-OCT, polarisation-sensitive optical coherence tomography; RNFB, retinal nerve fibre bundle; RNFL-S, retinal nerve fibre layer thickness slope; VF, visual field.

A polarisation-sensitive optical coherence tomography (PS-OCT) prototype was used to acquire volume scans on different retinal locations to stitch a wide-field volume covering approximately 45° of the fundus. Trajectories of the RNFBs were traced from the 24–2 and 10–2 VF test points to the optic disc (OD) and to their distal end using an adapted RNFB tracing method from Schwarzhans *et al*.^19^ Virtual B-scans along the traced RNFBs were then used to automatically segment the RNFL. The segmentation was checked manually and corrected if necessary. B-scans that showed an adjacent or crossing retinal blood vessel in the region close to the VF test point were excluded due to their effect on RNFL-S. The RNFL-S was calculated by performing a linear fit of a 0.6 mm-long RNFL segment from the virtual B-scan, centred on the corresponding VF point, using the polyfit function of MATLAB (The MathWorks, Inc., Natick, Massachusetts, USA) for a first-degree polynomial. The 0.6 mm segment on the retina is the anatomical correlate of the 2° separation between adjacent VF points in the 10–2 test, based on the established conversion factor of approximately 0.3 mm of retinal distance per degree of visual angle on the central macula. A positive RNFL-S indicates an increase in RNFL thickness towards the OD. Figure 1B illustrates an example of how virtual B-scans are extracted along individual RNFBs, and how the RNFL-S is subsequently determined from a 0.6 mm long segment centred on a VF test point.

VF tests were performed using the Humphrey Field Analyzer II (Carl Zeiss Meditec, Inc., Dublin, California, United States) with the Swedish Interactive Thresholding Algorithm (SITA-Standard, stimulus size III). All patients underwent two 24–2 and two 10–2 VF tests on two different days within 2 weeks. VFS was calculated as the average threshold (in dB) at each test location, derived from merged duplicate 24–2 and 10–2 fields VF test point locations, which were scaled and rotated to align with the wide-field PS-OCT volume using the coordinates of the blind spot and foveal centre from the VF, which were then matched with the corresponding ONH centre and foveal centre from the PS-OCT image. The positions of VF test points were then adjusted based on the model of Drasdo *et al*.^21^

Left eye data were mirrored to align with right eye, and group average maps were presented in field view.

## Statistical analysis

Continuous variables were compared between groups using t-tests. Point-wise Spearman correlation coefficients were calculated between RNFL-S and VFS. P values <0.05 were considered statistically significant.

To describe the influence of RNFL-S on VFS across the entire VF, a linear mixed-effects model (LMM) was fitted to analyse VFS, with fixed effects including glaucoma status, patient age, RNFL-S and axial length. A spatial correlation structure was incorporated into the model to account for the dependence between VF test point locations. An exponential function was identified as the best fit for the spatial correlation structure.

To account for the age difference when comparing RNFL-S between healthy and glaucomatous eyes, a generalised additive mixed model (GAMM) was fitted to analyse the relationship between RNFL-S, age and spatial location (X, Y). A smooth surface was used to model non-linear spatial effects and age, while glaucoma status was treated as a categorical variable. A random effect for each individual accounted for correlation within subjects.

Additionally, a point-wise LMM was fitted separately at each VF location to analyse the effect of RNFL-S, age and glaucoma status on VFS. Coefficients and corresponding t-test statistics were evaluated across locations.

## Results

**Figure 2:**
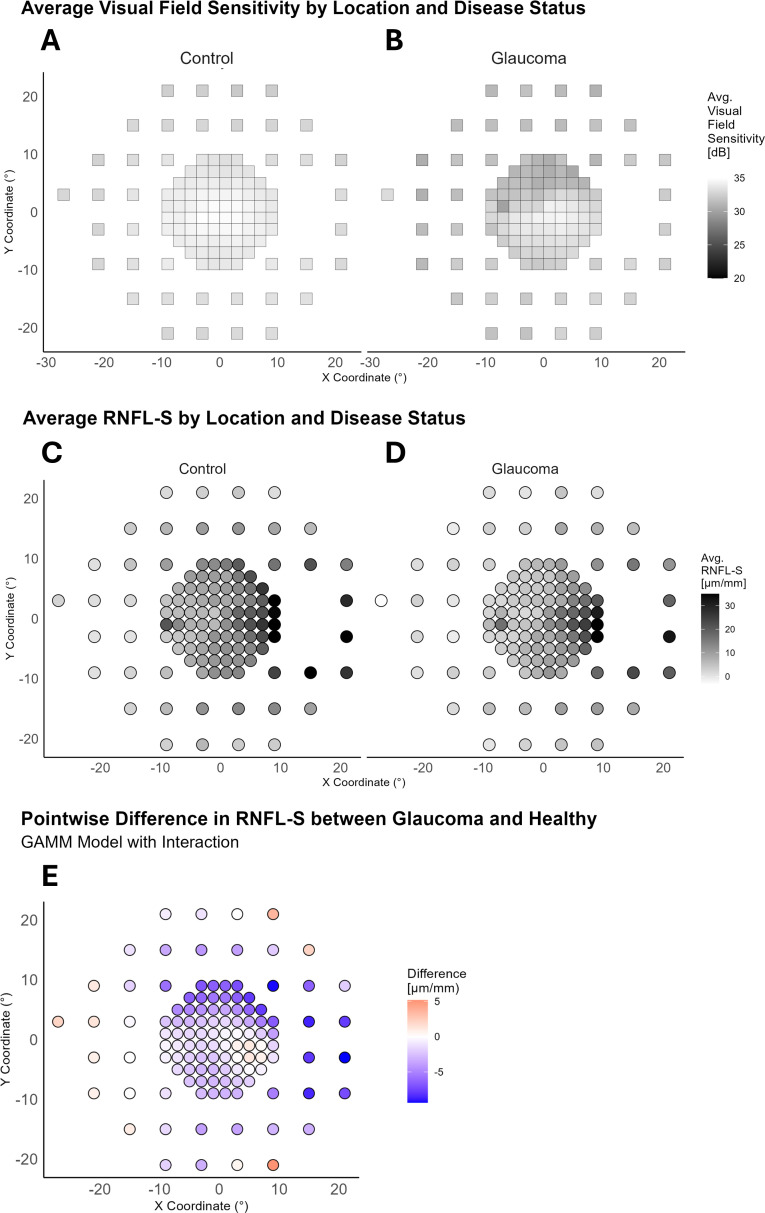
Averaged threshold values (dB) of glaucoma (A) and controls (B). Averaged RNFL-S maps in field view for healthy (C) and glaucomatous eyes (D) and the difference in RNFL-S between the two groups (E) accounting for age and spatial location of the VF test points using a GAMM. GAMM, generalised additive mixed model; model; RNFL-S, retinal nerve fibre layer thickness slope.

Subject characteristics are shown in table 1. The glaucoma cohort was significantly older compared with the controls. The averaged VF MD was −3.11±1.51 dB in glaucoma and −0.36±1.10 dB in healthy eyes. As can be seen in figure 2, VF defects were mainly observed in the superior hemisphere and in the nasal VF.

**Table 1:** Characteristics of study groups

Characteristics	Healthy	Glaucoma	P value
	n=139	n=50	
Age (years), mean±SD (range)	47.9±16.3 (21.1 to 78.9)	63.0±9.3 (39.9 to 77.6)	<0.001
Sex, n (%)			
Female	79 (57)	30 (60)	0.700
Axial eye length (mm), mean±SD (range)	23.7±1.0 (21.2 to 26.6)	24.0±1.0 (21.7 to 26.2)	0.119
Spherical equivalent (D), mean±SD (range)	−0.6±1.8 (−4.9 to 4.5)	−1.4±1.8 (−5.0 to 1.6)	0.022
Intraocular pressure (mmHg), mean±SD (range)	14.7±2.5 (9 to 20)	15.0±3.4 (8 to 23)	0.565
C/D ratio, mean±SD (range)	0.31±0.13 (0.00 to 0.70)	0.77±0.12 (0.30 to 0.95)	<0.001
VF MD 24–2 (dB), mean±SD (range)	−0.36±1.10 (−3.07 to 1.76)	−3.11±1.39 (−5.95 to −0.4)	<0.001
VF PSD 24–2 (dB), mean±SD (range)	1.54±0.33 (−2.30 to 2.40)	4.88±2.63 (−1.25 to 10.62)	<0.001
VF MD 10–2 (dB), mean±SD (range)	−0.44±1.01 (−2.50 to 1.78)	−2.92±3.82 (−13.79 to 1.10)	<0.001
VF PSD 10–2 (dB), mean±SD (range)	1.15±0.23 (0.83 to 1.78)	4.96±7.0 (1.11 to 14.98)	<0.001

C/D ratio, cup-to-disc ratio; MD, mean deviation; PSD, pattern SD; VF, visual field.

The overall mean RNFL-S per subject was less steep in glaucoma eyes compared with healthy (glaucoma: 9.5±3.1 µm/mm vs healthy: 12.5±2.3 µm/mm, p<0.001). In healthy subjects, VFS and the corresponding RNFL-S were 31.8 ± 2.4 dB (12.5 ± 2.9 µm/mm) in the upper hemisphere and 32.4 ± 2.2 dB (12.5 ± 2.5 µm/mm) in the lower hemisphere. In glaucoma patients, these values were significantly lower: 27.5 ± 7.0 dB (8.6 ± 3.9 µm/mm, p < 0.001) in the upper hemisphere and 30.1 ± 4.3 dB (10.5 ± 3.3 µm/mm, p < 0.001) in the lower hemisphere. The steepest RNFL-S was observed near the OD, while it was close to zero on the more peripheral VF points and near the temporal raphe. When healthy and glaucoma patients are combined, the Spearman correlation between averaged RNFL-S and VFS was r=0.469 for the upper hemisphere and r=0.290 for the lower hemisphere. Raw data of RNFL-S and VFS are shown in the supplemental materials (online supplemental figure S1).

Figure 2C, D shows the average RNFL-S for healthy and glaucoma eyes. Figure 2E shows the difference in predicted RNFL-S between the two groups accounting for age and spatial location of the VF test points using a GAMM. The GAMM demonstrated statistical significance, explaining 49.9% of the RNFL-S (R²=0.499). The presence of glaucoma was significantly associated with a lower RNFL-S (β=−2.6774, SE=0.4077, p<0.001). The spatial location (X, Y) had a strong influence on the RNFL-S, indicating that the RNFL-S varies across different retinal regions. Age also had a significant effect, with older individuals showing lower RNFL-S values. Additionally, there was substantial variability between individuals. The axial length was not included in the model as it had no significant influence on RNFL-S. The difference in predicted RNFL-S values shown in figure 2E confirmed that glaucoma patients had lower RNFL-S values than controls.

An LMM was applied to investigate the relationship between VFS and the predictors RNFL-S, glaucoma status and age, considering the entire dataset. The model demonstrated statistical significance, explaining 18.9% of the variance in the VFS (R²=0.1885). A positive association with VFS was observed for RNFL-S (β=0.0415, SE=0.0023, t=17.68, p<0.001). Presence of glaucoma was significantly associated with a lower VFS (β=−2.468, SE=0.069, t=−35.70, p<0.001). Age was negatively associated with VFS (β=−0.048, SE=0.002, t=−25.04, p<0.001).

**Figure 3:**
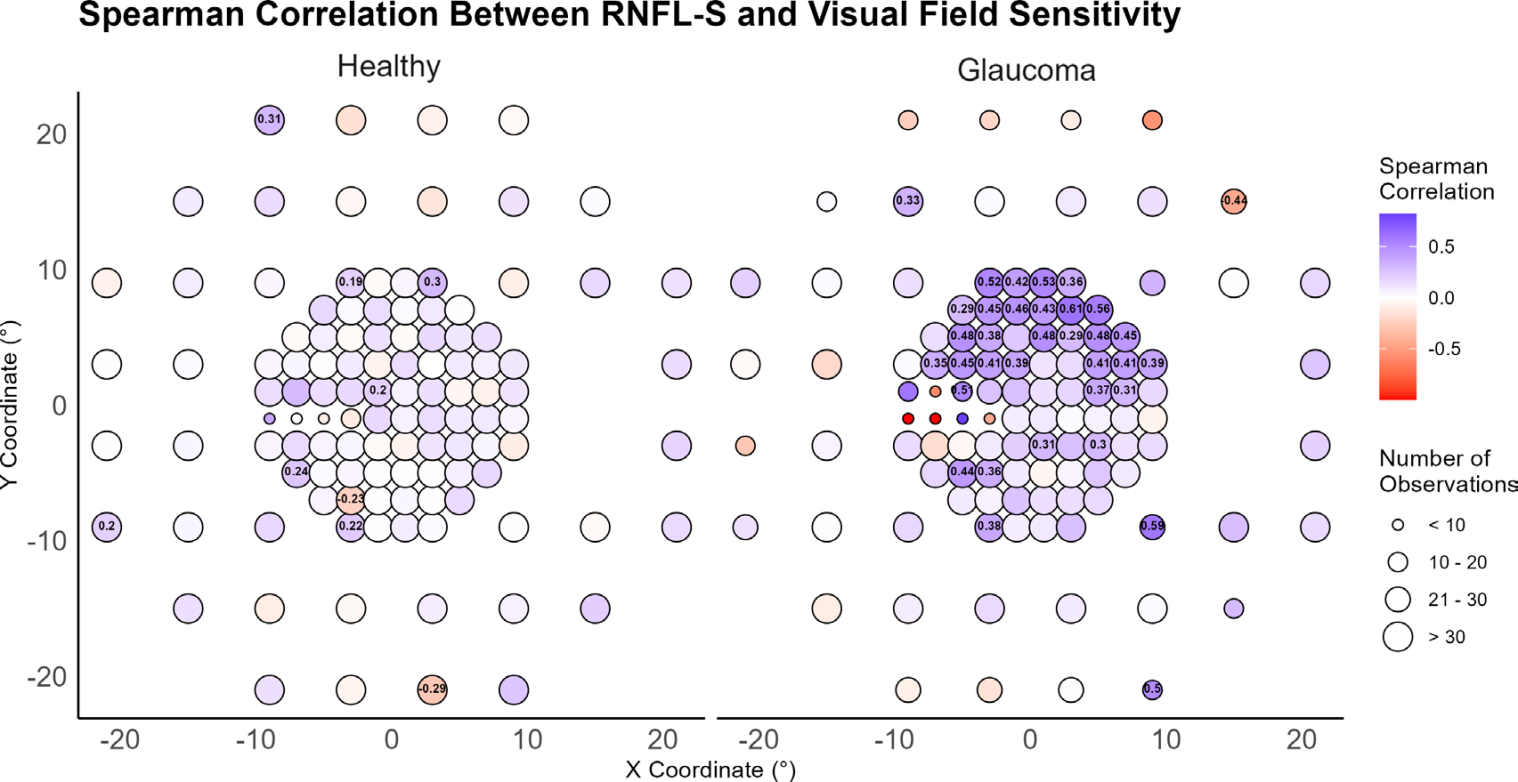
Point-wise Spearman correlations between RNFL-S and visual field sensitivity in healthy and glaucoma subjects. Numeric values shown only for significant correlation coefficients, p<0.05. RNFL-S, retinal nerve fibre layer thickness slope.

Point-wise Spearman correlation coefficients between VFS and RNFL-S are shown in figure 3. In glaucoma eyes, the highest Spearman correlations were observed in the upper hemisphere within the central 10° and nasally near the ONH, specifically in areas with the most frequently observed VF defects, with significant correlations reaching up to r=0.61. The median of significant correlations in glaucoma eyes was r=0.42 (IQR: 0.37–0.48), and for all correlation coefficients (including non-significant values), the median was r=0.25 (IQR: 0.10–0.41). In healthy eyes, significant correlations showed a median of r=0.21 (IQR: 0.19–0.24), while for all coefficients (including non-significant ones), the median was r=0.05 (IQR: 0.00–0.14). At points with fewer observations, outliers were noted. However, these were not statistically significant.

**Figure 4:**
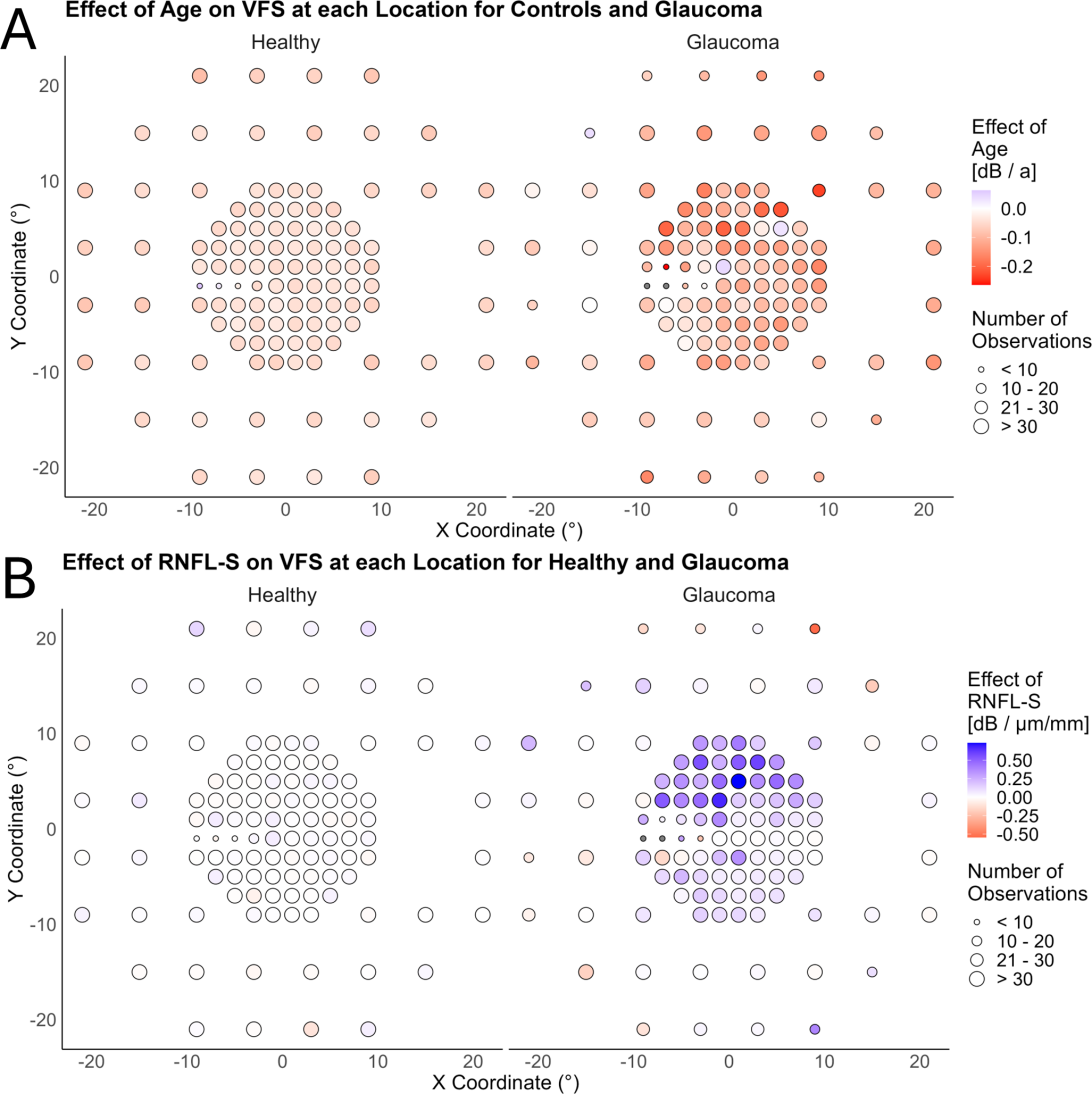
Point-wise models exhibiting the influence of age (A) and RNFLS (B) on VFS in healthy (left) and glaucomatous eyes (right). RNFL-S, retinal nerve fibre layer thickness slope; VFS, visual field sensitivity.

Figure 4 illustrates the location-specific influence of age (figure 4A) and RNFL-S (figure 4B) on VFS for healthy (left) and glaucomatous eyes (right). A linear model was fitted separately at each VF location to analyse the effect of RNFL-S, age and glaucoma status on VFS. As can be seen in figure 4 (upper plot), the effect of RNFL-S is particularly pronounced at the locations where the lowest VFS was observed in glaucomatous eyes. In contrast, little to no effect of RNFL-S was observed in healthy eyes. In areas with very few observations, an unreliable effect in the opposite direction was found. When examining the effect of age (see figure 4 lower plot), a weak negative correlation between age and VFS can be detected in healthy eyes.

## Discussion

The relationship between structural and functional changes in glaucoma is a cornerstone of understanding and managing the disease. Structural measures, derived from OCT, have provided significant advancements in detecting glaucomatous damage.^22^ However, the challenge lies in bridging these structural changes with functional outcomes, as measured by VF tests. Within the macular area, where the retinal ganglion cell (RGC) layer is thick and can be measured by OCT, measures of the RGC layer have been used for structure–function correlations.^23^,^25^ Outside the macular area, measures of the local RGC count are still lacking.

Based on our assumption that RNFL-S represents a local measure of the count or density of ganglion cell axons entering the RNFL, our study demonstrates that RNFL-S can serve as an indicator of visual function. Nevertheless, as with other structure–function models,^2 13 14 26 27^ we were unable to achieve a perfect correlation. The factors contributing to this are varied, with one being the considerable variability of RNFL-S across the fundus. RNFL-S exhibits its highest values near the optic nerve and in the region of the major arcuate vessels. Consequently, when comparing RNFL-S changes to VF function, it is essential to acknowledge the non-uniform distribution of RNFL-S across the eye.

One reason for the uneven distribution is the fact that retinal nerve fibres converge while approaching the ONH, leading to a convergence effect. This convergence may contribute to intersubject and intrasubject variability in RNFL-S measurements and could partially obscure local correlations with visual function, depending on the retinal location. A further effect that might influence RNFL-S is the presence of nearby or intersecting vessels in the region of the measurement. In addition, variations in ganglion cell axon diameters and densities across the fundus might result in shallower or steeper RNFL-S values.

Existing structure–function models are largely based on the correlation of RNFL thickness, ONH parameters, ganglion cell count, GCL thickness, ganglion cell complex (GCL+inner plexiform layer (IPL)), the IPL or OCT–angiography parameters. Models that take the peripapillary RNFL thickness into account, such as the well-known mapping of the VF to the OD by Garway-Heath *et al*,^23^ have the advantage that peripapillary RNFL measurements allow predictions about VFS in specific areas of the VF. This simplifies analysis by enabling a direct comparison between VF defects and damaged RNFL, ultimately improving diagnostic accuracy.^28^ However, a disadvantage of this approach is that while this mapping may be valid for many patients, there is significant interindividual variability in the trajectories of nerve fibres, which can render the model inaccurate in certain cases.^29^ This variability can be reduced through individualised structure–function maps.^30^,^32^ In our analysis, we were able to trace individual nerve fibre bundles, allowing us to develop and evaluate a local measure that is thought to represent the local ganglion cell population at each VF point location. This was corroborated by correlations with the VFS.

Our model aligns more closely with point-wise ganglion cell-based models. In these models, VF points are mapped onto the retina, typically using the model by Drasdo *et al*^21^ or other displacement methods, to account for the displacement of ganglion cells from foveal cones. The uncertainty inherent in this approach stems from the potential discrepancy between the mapped VF points and their actual corresponding retinal locations at the level of the RGCs. Nevertheless, studies have demonstrated that this discrepancy is likely relatively small.^33^ In our study, we used the Drasdo *et al* model^21^ and implemented individual tracing to minimise this effect. This approach enabled us to present a novel potential measure of the local RGC count, allowing such measurements outside the central 6°–8° degrees of the VF.

Although our study highlights the potential of RNFL-S as a biomarker for structural glaucoma damage and the structure–function relationship, it is important to evaluate its performance in relation to other structure–function correlation methods.

Advanced imaging technologies have introduced numerous metrics to bridge the structure–function gap, with varying success in correlating with VFS. Some studies have reported correlations up to r=0.81 between sectoral peripapillary RNFL and averaged VF sensitivities, typically in cohorts with more advanced glaucoma than ours.^4 5 27^ In studies focusing on the central VF and its relationship with ganglion cell-related parameters, reported maximum correlations ranged from r=0.47 to r=0.706.^3 34 35^ To enhance correlation strength, these studies often averaged regions in both structural and functional maps before analysis, effectively smoothing the data. Reported differences in correlation values may arise from substantial methodological variability, including differences in study populations (glaucomatous vs healthy eyes), OCT modalities (eg, Spectralis vs Cirrus^4 5 27^), VF point clustering methods (eg, Garway-Heath sectors^23^) and individualised models such as that of Jansonius *et al*^17 18^ used for RNFL structure–function mapping. Correlations also vary depending on whether ganglion cell plus IPL (GC+IPL) thickness or estimated RGC count was used,^3 34 35^ and they tend to be higher in studies including participants with more severe VF loss.^4 34^

In multivariate analyses, combining multiple structural parameters has shown superior correlations compared with single metrics. For example, integrating the superficial vascular complex and RNFL achieved correlations up to r=0.710, compared with r=0.578 for RNFL alone and r=0.627 for superficial vascular complex capillary density.^15^ Similarly, we consider our presented method as a first step toward developing a more comprehensive model that incorporates RNFL-S alongside direct measures of RGC layer thickness (particularly within the central 6°s) and potentially the peripapillary RNFL. Despite relying on point-wise correlations, our study still demonstrated moderately strong Spearman correlations of up to r=0.61, aligning well with existing models. The strongest correlations were observed within the central 10° and near the OD, particularly in regions showing the most pronounced VF defects.

Although the LMM explained only 18.9% of the variance in VFS (R²=0.1885), this relatively low value can be attributed to the inclusion of all VF locations, including unaffected regions in both healthy eyes and early glaucoma cases. Interindividual variance at locations without VF defects is largely driven by measurement imprecision, which, as expected, reduces the proportion of variance explained by the LMM. Nevertheless, pointwise analysis revealed moderate local correlations between RNFL-S and VFS, particularly in areas with more frequent VF defects, highlighting the potential of RNFL-S as a regional biomarker of structural integrity in glaucoma. However, it should be noted that this correlation was significant only in certain regions with VF defects and not across the entire VF. While this localised finding underscores the regional nature of the structure–function relationship in glaucoma, it also limits the interpretation of the overall association between RNFL-S and visual function. Furthermore, RNFL-S was not directly compared with other established OCT-derived parameters, such as RNFL or GC+IPL thickness, which would provide valuable context for its relative performance. While our study provides evidence for the value of the RNFL-S, it is essential to acknowledge its limitations. Our findings are based on a specific patient cohort, and their generalisability to diverse populations requires further validation. Because this study used a cross-sectional design, we can identify associations between variables but cannot establish cause-and-effect relationships. There was a substantial age difference between the glaucoma and control cohorts, which we accounted for as a covariate in all statistical models. Nevertheless, such an imbalance may introduce residual confounding and should be considered when interpreting the results. We initially expected age to exert a similar influence on VFS in both healthy and glaucomatous eyes. However, as shown by figure 4A, we observed a stronger age-related effect in the glaucoma group. This might reflect the observed imbalance in VFS across the age range in our glaucoma cohort. As shown in online supplemental figure S2, the glaucoma group demonstrated a negative correlation of VF MD and age. Because we selected only patients with early glaucoma damage, the nature of this association remains speculative. Additionally, the anatomical convergence of retinal nerve fibres toward the ONH contributes to considerable intrasubject variability in RNFL-S within a single fundus, but might also contribute to intersubject differences. Addressing this effect through potential correction methods may improve correlation. Our model is most effective for VF points within the central 10° and near the OD, while its applicability to peripheral points is limited, as RNFL-S tends to approach zero in these areas. Another limitation is that some data points lacked sufficient observations due to the exclusion of incomplete RNFLB traces, potentially affecting the robustness of our findings. Furthermore, our approach relies on an automated individual tracing method, which was verified by a human expert. However, there may still be deviations from the actual RNFB trajectories, introducing a potential source of error. It is also important to note that RNFL-S was derived from a PS-OCT prototype, which is not yet available in clinical practice. Nevertheless, this method could be adapted to conventional OCT using existing RNFL bundle tracing models, such as the one developed by Jansonius *et al*,^17 18^ highlighting its potential for routine clinical application.

Future research should also explore the value of RNFL-S data in predicting ganglion cell density or count. Moreover, it could serve as a useful addition to multivariate models when combined with other imaging modalities, such as structural data from conventional OCT, PS-OCT, adaptive optics OCT or OCT angiography. This approach may further enhance our understanding of structure–function relationships in glaucoma and provide deeper insights into retinal architecture.

## Conclusion

We presented a first assessment of a structural–functional relationship represented by RNFL-S and VFS. Moderate correlations were observed in areas with VF defects. This suggests RNFL-S as a promising biomarker for focal glaucoma damage. However, larger cohorts and advanced analysis are needed for full evaluation.

## Supplementary material

10.1136/bjo-2025-328330 online supplemental figure 1

10.1136/bjo-2025-328330 online supplemental figure 2

## Data Availability

Data are available upon reasonable request.
