# Supplementary figures and images for: The retinal nerve fibre layer thickness slope: a localised biomarker of the structure–function relationship in early glaucoma
Source: Br J Ophthalmol. 2025 Dec 24;110(7):e328330. doi: 10.1136/bjo-2025-328330 (PMC13311959; doi:10.1136/bjo-2025-328330)

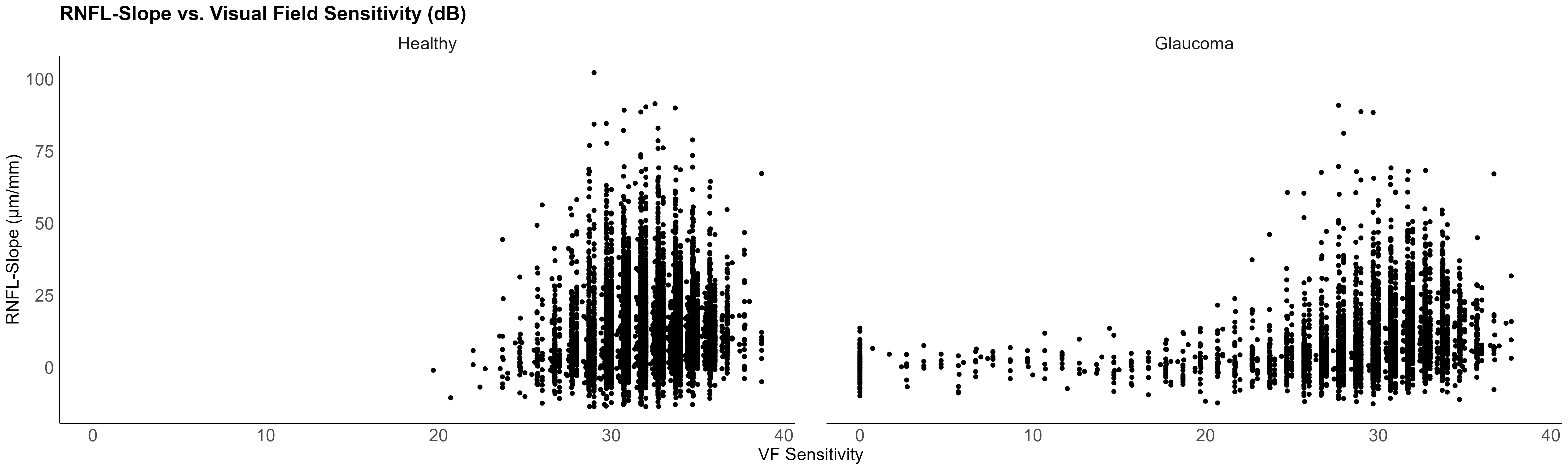

Supplement: online supplemental figure 1 [file bjo-110-7-s001.tiff]

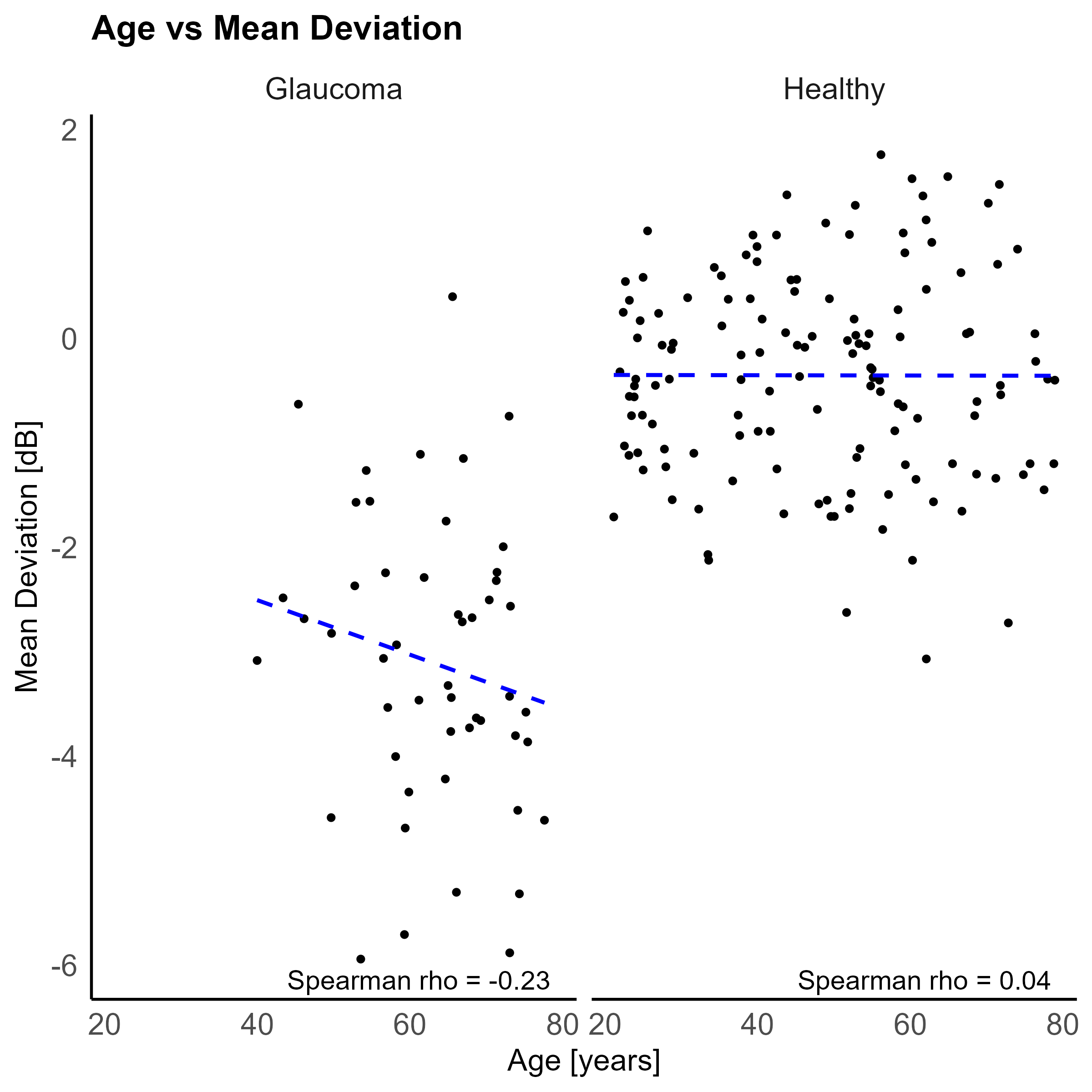

Supplement: online supplemental figure 2 [file bjo-110-7-s002.tiff]
